# Bagging Ensemble of Multilayer Perceptrons for Missing Electricity Consumption Data Imputation

**DOI:** 10.3390/s20061772

**Published:** 2020-03-23

**Authors:** Seungwon Jung, Jihoon Moon, Sungwoo Park, Seungmin Rho, Sung Wook Baik, Eenjun Hwang

**Affiliations:** 1 School of Electrical Engineering, Korea University, 145 Anam-ro, Seongbuk-gu, Seoul 02841, Korea; jsw161@korea.ac.kr (S.J.); johnny89@korea.ac.kr (J.M.); psw5574@korea.ac.kr (S.P.); 2 Department of Software, Sejong University, 209 Neungdong-ro, Gwangjin-gu, Seoul 05006, Korea; smrho@sejong.edu (S.R.); sbaik@sejong.ac.kr (S.W.B.)

**Keywords:** missing-value imputation, electric energy consumption data, smart meter, deep learning, multilayer perceptron, ensemble learning

## Abstract

For efficient and effective energy management, accurate energy consumption forecasting is required in energy management systems (EMSs). Recently, several artificial intelligence-based techniques have been proposed for accurate electric load forecasting; moreover, perfect energy consumption data are critical for the prediction. However, owing to diverse reasons, such as device malfunctions and signal transmission errors, missing data are frequently observed in the actual data. Previously, many imputation methods have been proposed to compensate for missing values; however, these methods have achieved limited success in imputing electric energy consumption data because the period of data missing is long and the dependency on historical data is high. In this study, we propose a novel missing-value imputation scheme for electricity consumption data. The proposed scheme uses a bagging ensemble of multilayer perceptrons (MLPs), called softmax ensemble network, wherein the ensemble weight of each MLP is determined by a softmax function. This ensemble network learns electric energy consumption data with explanatory variables and imputes missing values in this data. To evaluate the performance of our scheme, we performed diverse experiments on real electric energy consumption data and confirmed that the proposed scheme can deliver superior performance compared to other imputation methods.

## 1. Introduction

A typical energy management system (EMS) is equipped with smart meters (SMs) that measure the amount of electric energy consumed [1,2]. These meters collect such information from diverse targets such as houses, buildings, and cities, and the EMS performs appropriate operations based on the information. For instance, future electric energy consumption can be predicted based on the collected data. Thus, collecting precise SM data is essential because the forecasting accuracy significantly depends on the quality of collected data [3,4]; further, accurate energy consumption forecasting enables the EMS to perform energy management efficiently and effectively [5].

However, guaranteeing accuracy in the energy consumption data is not trivial because there are several factors that result in missing data [6,7]. For example, malfunctions of the device and signal transmission errors are typical sources of missing data [8]. This missing value problem decreases the prediction accuracy and results in inferior performance for the forecasting methods that are based on consecutive values, such as the autoregressive integrated moving average [9,10]. A popular approach to resolving this problem is to replace the missing values with plausible values, which is known as missing-value imputation.

Several missing-value imputation methods have been proposed for resolving the missing electric energy consumption data. One intuitive method involves the use of interpolation with temporally adjacent data. Interpolation is a method of generating new data points located between known data points. A linear interpolation (LI) is a typical example of interpolation that exploits a straight line. If missing data points are identified, LI creates a line connecting two known data points just outside the missing data interval; then, the missing data points are replaced with data points derived from the line. LI is relatively easy to use when compared to other imputation methods. However, it demonstrates the limitation of inaccurate prediction when the length of the missing data interval is long. Further, the method of using historical data is also well known. If a missing data point is identified, this method searches for potential data points in the historical data and imputes the missing data point. A historical average (HA) model is an example of such a method that replaces the missing data point with an average of a few historical data points having similar properties such as time and location. Similarly, there are methods based on the nearest neighbor (NN). These methods determine similar patterns in historical data by considering the NN data points near the missing data point and replacing the missing data point with the data point determined by the identified pattern. Such methods based on historical data can deliver superior performance when the entire data set has a consistent pattern. However, the imputation result considerably depends on the previous data; consequently, these methods may fail if an abrupt change occurs in the data pattern.

Recently, certain studies were conducted to overcome the aforementioned problems. Peppanen et al. [7] proposed an optimally weighted average (OWA) method that combines both the LI and the HA. If the length of the missing data interval is larger than the predefined threshold, the imputation result is primarily effected by the HA, and if not, it is effected by the LI. This method avoided the drawbacks of both the LI and the HA; therefore, it delivered better accuracy than those of the two individual methods. Kim et al. [8] introduced a learning-based adaptive imputation (LAI) method based on feature vectors of the data points surrounding the missing data point. By utilizing intentionally generated missing data, the LAI method learns the optimal length of the feature vector and the range of the historical data that must be considered during the imputation. Then, it calculates the distance between feature vectors, selects k most similar past situations according to the distances and estimates the missing data point. Wang et al. [11] proposed a method of adopting a weighted summation of results from both linear regression (LR) and NN methods. The weight for yielding the outcome depends on the errors of the two models, which makes the imputation result almost similar to the output from the model with a lower error. Moghaddass and Wang [12] resolved the missing value problem in the smart grid system by modeling the data using probabilistic distributions. They represented the data and error occurrence using Poisson and Bernoulli distributions, respectively, and selected the estimated average to impute missing values. Grigoras et al. [13] proposed two methods for handling missing data of load profiles; one using k-nearest neighbor and the other using clustering with a fuzzy model. They found values with patterns similar to those around missing values by using those methods and replaced the missing values with the values voted most by the selected patterns. Huang et al. [14] suggested an energy consumption data-imputation model using the relationships between energy consumption data and various factors, such as voltage, resistance, and time. Their model consisted of small models representing the correlation between energy consumption and each of the factors. According to the conditions, suitable models were selected among the small models and utilized to impute the missing data point. Turrado et al. [15] presented a self-organized maps neural network-based method using several factors, such as each phase current, voltage from phase to phase, and voltage from phase to neutral. They constructed a data matrix using those factors and extracted director vectors using the self-organized maps neural network. Then, they calculated distances between director vectors and imputed missing values by the director vector with the lowest distance.

Unlike the studies conducted previously, we considered the approach of using a machine learning algorithm with explanatory variables. This approach can be categorized as a regression imputation [16] approach; moreover, it is possible to achieve remarkable performance according to the selected variables and the regression model used [17,18]. It must be noted that this approach is not suitable for a real-time environment owing to the requirement of additional variable collection and model training. However, we have primarily focused on achieving an accurate imputation to improve energy consumption forecasting; therefore, this drawback is not considered in this study.

In this study, we propose the use of a multilayer perceptron (MLP). An MLP is one of the artificial neural network models that imitates a human brain heuristically. Recent studies based on the MLP have demonstrated that it can outperform other machine learning algorithms in various fields, including regression models [19,20]. Thus, for better accuracy, we decided to apply an ensemble of MLPs in the proposed method. An ensemble implies the application of more than one learning algorithm. An ensemble is known to capture the uncertainty better than when only one algorithm is used; consequently, it can achieve superior performance [21]. However, the performance of the ensemble is highly dependent on the method used to combine the submodels [22]. The most common method adopts an average result of all the submodels; however, we introduce another method of combining the MLPs.

We propose an imputation scheme based on the softmax ensemble network (SENet) which is a bagging ensemble of MLP subnetworks. With several explanatory variables, SENet performs missing-value imputation by considering the outputs and weights of MLP subnetworks. This model is trained in two stages. In the first stage, each subnetwork is independently trained by the dataset allocated to it. In the second stage, the ensemble weight assigned to each subnetwork is adjusted, and unnecessary subnetworks are removed based on the ensemble weight. These two stages are repeated until the convergence criterion is satisfied or the number of iterations reaches a predefined number. Consequently, our scheme offers better missing data-imputation accuracy than the conventional methods. To prove the effectiveness of our scheme, we performed several experiments by using real electric energy consumption data, and we report the results later.

The contributions of this study are as follows:

1. We introduce an imputation scheme using explanatory variables. The explainable variables such as weather and calendar are useful in improving the imputation accuracy.
2. We introduce a novel ensemble model based on several MLPs and a softmax function. Further, we apply random sampling with replacement to enhance the ensemble effect.
3. Based on the experiments using real electric energy consumption data, we report that the proposed scheme can deliver better performance than those of other missing-value imputation methods.

This paper is organized as follows. In Section 2, we present the overall architecture of our model and the training process. We present the results of the performed experiments in Section 3. Finally, we conclude the study in Section 4.

## 2. Methods 

**Figure 1:**
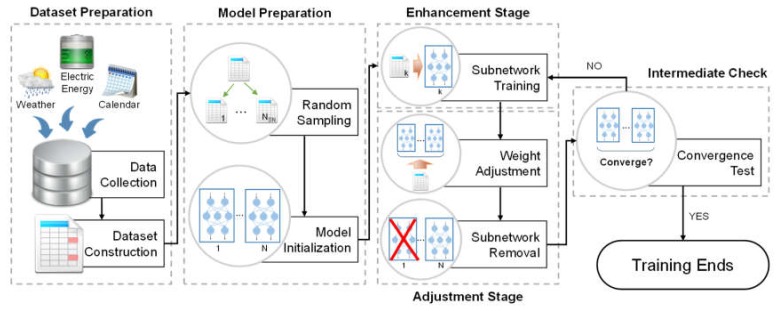
Overall process of the proposed scheme.

The overall process of the proposed scheme is shown in Figure 1. The scheme consists of five steps: dataset preparation, model preparation, enhancement stage, adjustment stage, and intermediate check. We subsequently describe each step in detail.

### 2.1. Dataset Preparation

**Figure 2:**
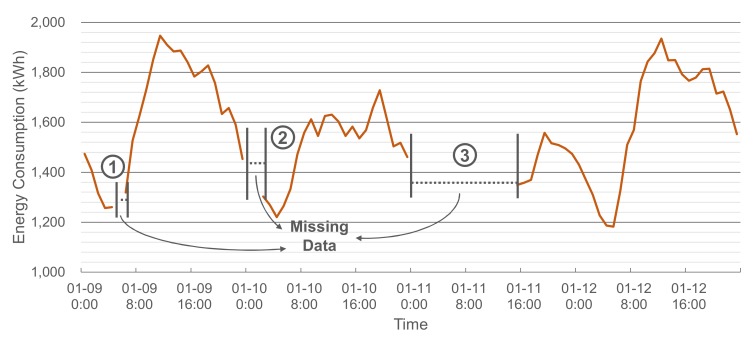
Example of the energy consumption data with missing data intervals.

The proposed scheme is based on the ensemble model with several MLPs. In the first step, data collection and dataset construction are performed for training the ensemble model. The electric energy consumption data are collected from a SM, which becomes the output variable of the ensemble model; i.e., the imputation target. Figure 2 illustrates an example of the collected energy consumption data with three missing data intervals. In the figure, the length of the third interval is relatively long compared to the other two intervals. For such a long interval, imputations based on temporally adjacent data tend to give poor performance.

For input variable configuration, the explanatory data, such as weather and calendar data, are collected from diverse data sources, such as energy management systems and web sites. The periods of these collected data should be the same. For instance, if the SMs record energy consumption per hour, the explanatory data also should be measured per hour. 

In the weather data, temperature, humidity, wind speed, temperature humidity index (THI) and wind chill index (WCI) are included. As these factors are used practically for forecasting the energy consumption, they were selected as the input variables [23,24]. The calendar data contains information on the measurement time, such as a timestamp, the working schedule of the management target and holiday dates.

The calendar data requires preprocessing. First, we determined the season, month, day, hour, minute, and day of the week through the timestamp. Here, the minute is not considered if the measurement period is equal to or longer than 1 h. Second, the time units, such as a month, day, and hour, were preprocessed to reflect the periodicity [5,23] using Equations (1) and (2), where *time* is the time unit we want to transform and *period_time_* is its period. For example, *period_time_* will be 12 if we convert the month, while *period_time_* will range from 28 to 31 if we convert the day of the month. Third, season and day of the week are represented by one-hot vectors. Finally, two variables are added to indicate whether the measurement time is obtained on a working hour of the management target or on a holiday. After the calendar data is preprocessed, 26 input variables are prepared; these variables and the output variable are listed in Table 1.

(1) $$time_{x}=\sin\left(\left(\frac{2\pi }{period_{time}}\right)\times time\right)$$

(2) $$time_{y}=\cos\left(\left(\frac{2\pi }{period_{time}}\right)\times time\right)$$

**Figure 3:**
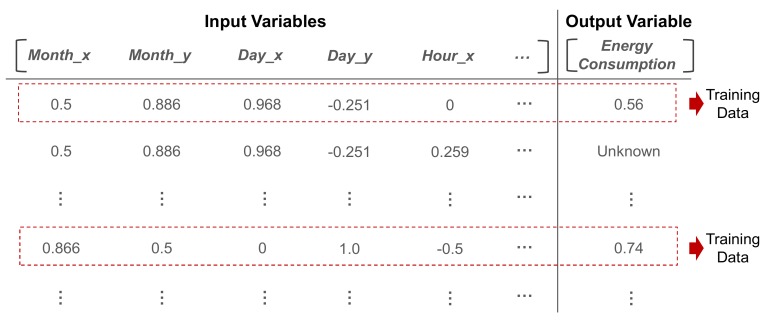
Example of the portion of the entire dataset.

To fit the range of all the variables, we applied min-max normalization to ensure that these variables ranged from zero to one. Thus, the entire dataset is obtained as shown in Figure 3; moreover, among all the data points, the data points for which the value of the output variable is known will be used for training the ensemble model.

### 2.2. Model Preparation

For the construction of the ensemble model, we determined the number of subnetworks that comprise the ensemble model, denoted by *N*. The probability of obtaining a better model at the end of the training increases as the number of subnetworks in the model increases. However, the training time becomes significantly longer corresponding to the increase in *N*. Empirically, we showed that the proposed model can achieve reasonable results when *N* is set equal to or more than 20.

After N is set, random sampling with replacement is conducted. The sampling generates N different datasets; moreover, each subnetwork possesses its own dataset. Thus, the subnetwork is trained by its dataset and yields a variety of prediction results that strengthen the ensemble effect. This method is commonly called either bootstrap aggregating or bagging. A famous example of bagging is random forest (RF) [25]. RF is a tree-based ensemble model that uses bagging to grow various trees. Its outstanding generalization performance and accurate prediction performance have been reported in several works [26,27], which motivated us to adopt bagging for our model.

**Figure 4:**
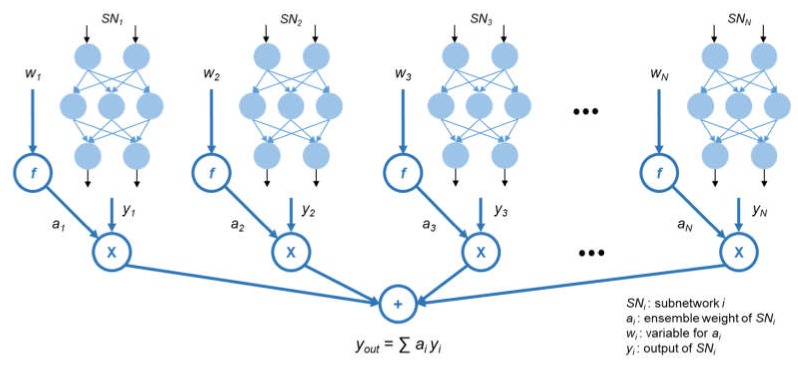
Structure of SENet.

Figure 4 shows the structure of the ensemble model. *N* subnetworks are created after the sampling, and each subnetwork *SN_i_*, *i* = 1, 2, 3, …, *N*, additionally has an ensemble weight *a_i_*. *a_i_* is used to calculate the result of the ensemble model, *y_out_*, which is a weighted average of the output of each subnetwork *y_i_*. However, for ensuring that *y_out_* is the weighted average, the ensemble weights must satisfy the following two conditions: (1) all ensemble weights must be contained in the interval from zero to one (*a_i_* ∈ [0, 1]); (2) the sum of the ensemble weights must be one (∑*a_i_* = 1). While designing the deep learning model, it is difficult to identify a method to train the model while satisfying these two conditions. However, a softmax function used primarily in classification tasks always satisfies these conditions. Hence, we decided to apply a softmax function and adopt a new variable *w_i_* to determine *a_i_*. The softmax function is defined as Equation (3). Owing to the property of a softmax function, the ensemble weights always satisfy these two conditions. Therefore, in the model training, *a_i_* is not directly adjusted; however, *w_i_* is adjusted instead of *a_i_*. The change of *w_i_* affects *a_i_* such that we can determine the proper value of *a_i_*. In the remaining sections of this paper, we express that we directly adjust *a_i_* for convenience.

(3) $$a_{i}=f\left(w_{i}\right)=\frac{e^{w_{i}}}{\sum_{k=1}^{N}\left(e^{w_{k}}\right)}$$

In this model, there are two parts that should be optimized: (1) the subnetworks and (2) the ensemble weights. Initially, all the parameters, including parameters of the subnetworks and the ensemble weights, are assigned to arbitrary values. The former is optimized in the enhancement stage, while the latter is optimized in the adjustment stage.

### 2.3. Enhancement Stage

In this stage, each subnetwork is trained independently. The loss of *SN_i_* in the enhancement stage, *loss_enh,i_*, is defined according to Equation (4).

(4) $$loss_{enh,\;i}=E\left[{\left(t-y_{i}\right)}^{2}\right]$$

Here, *t* is the real electric energy consumption data and *y_i_* is the estimated energy consumption by *SN_i_*. Without interfering with each other, all subnetworks learn their own datasets to minimize the loss function. Meanwhile, the ensemble weights are fixed and unable to affect the training in this stage. If all subnetworks are trained *Epoch_enh_* times, this stage is completed, and the next stage, i.e., the adjustment stage, commences.

### 2.4. Adjustment Stage

In this stage, the ensemble weights are adjusted, and unnecessary subnetworks are removed based on these weights. Unlike the enhancement stage, only the ensemble weights are changeable during this training, while all the parameters of the subnetworks are fixed. That implies that *y_i_* cannot be changed in this stage. Simply speaking, this process can be considered as though we train a shallow artificial neural network whose input variables are the outcomes from subnetworks and parameters are the ensemble weights. The loss of adjustment stage, *loss_adj_*, is calculated according to Equation (5).

(5) $$loss_{adj}=E\left[{\left(t-y_{out}\right)}^{2}\right]=E\left[{\left(t-\sum_{i=1}^{N}a_{i}y_{i}\right)}^{2}\right]$$

The dataset used for the training is the original dataset before the random sampling with replacement. The reason for this is overfitting. While training each subnetwork, there is no requirement to be concerned about overfitting because the ensemble model can avoid overfitting by controlling the ensemble weights. However, if we use only a randomly sampled dataset for adjusting the ensemble weights, it degrades the accuracy owing to a higher possibility of the occurrence of overfitting. Therefore, all available data points are utilized.

After the training in this stage is performed *Epoch_adj_* times, we examine whether the ensemble weight of each subnetwork is less than the predefined threshold *α_th_* or not. If the weight is less, this subnetwork will be removed from the ensemble model. This step is performed as the subnetworks having low ensemble weights rarely contribute to accurate predication, when compared to those having larger ensemble weights. Thus, it is preferable to remove them to reduce the training time than to retain them.

For example, let us assume that *a_1_* = 0.01, *a_2_* = 0.05, *a_3_* = 0.15, and *a_4_* = 0.79. When we set *α_th_* = 0.02, only *SN_1_* will be deleted. If *α_th_* = 0.1, both *SN_1_* and *SN_2_* will be removed. *α_th_* affects the number of subnetworks remaining after the training. Thus, if we require a light ensemble model, we can set *α_th_* to be high. However, significant accuracy degradation might be observed when *α_th_* is too large.

The next step for the remaining subnetworks depends on the intermediate check. That is, if the intermediate check indicates continuous training, the enhancement stage restarts for the remaining subnetworks; otherwise, the training process stops and the ensemble of the remaining subnetworks is exploited as an imputation model. 

### 2.5. Intermediate Check

After the subnetwork removal, the intermediate check is performed. If the ensemble model satisfies the condition, the model training will be concluded, and missing-value imputation is performed using the trained ensemble model; otherwise, the enhancement stage restarts. Until the number of iterations of the two training stages reaches *N_MAX_*, an intermediate check is conducted every time the adjustment stage is completed. The goal of the intermediate check is to confirm whether the ensemble model requires no further training. As the model training is more time consuming in comparison with other machine learning algorithms, the intermediate check is required to shorten the total time consumption of the model training.

**Figure 5:**
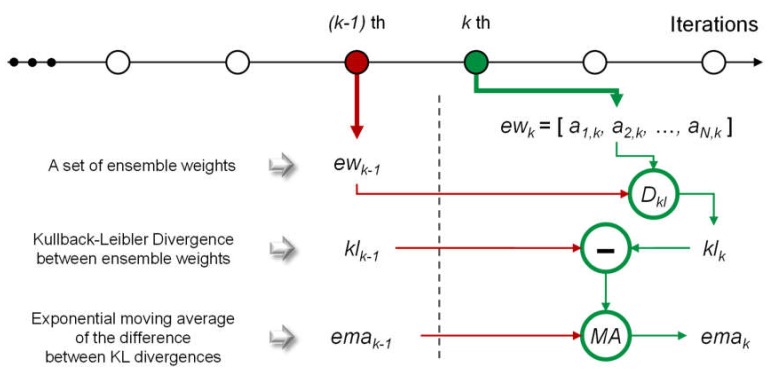
Flow required for the calculation of *ema_k_.*

Here, we explain the condition that is checked in this stage. As machine learning models do not require more training when they converge, we assume that the ensemble weights are not changed if the models converge. The calculation flow for checking is described in Figure 5. Here, we denote all ensemble weights at the *k*th iteration of the training stages as *ew_k_* = [*a_i,k_*, *a_2,k_*, …, *a_N,k_*]. As described in Section 2.2, the ensemble weights satisfy the two conditions owing to the properties of the softmax function, which also follows the properties of a probability distribution. Thus, we treat *ew_k_* similarly to a probability distribution. By the given assumption, when the ensemble model converges, there is no difference between *ew_k_* and *ew_k−1_*. To measure the difference, we use a Kullback–Leibler divergence (KLD), which is a measure of the difference between two probability distributions. KLD will be low when the given probability distributions are similar, and KLD becomes zero if the two probability distributions are exactly the same. Conversely, KLD will be large when the probability distributions vary significantly. Therefore, we should determine *k* when the KLD value between *ew_k−1_* and *ew_k_*, denoted by *kl_k_*, is zero. Equation (6) shows the equation of *kl_k_*, where *D_kl_*(*x*||*y*) indicates KLD between two probability distributions *x* and *y*.

(6) $$kl_{k}=D_{kl}(ew_{k-1}||ew_{k})=\sum_{i=1}^{N}a_{i,k-1}\times \log\left(\frac{a_{i,k-1}}{a_{i,k}}\right)$$

However, it is impractical to observe a case wherein *kl_k_* becomes zero. In practice, *kl_k_* generally converges on a specific value, not zero. Consequently, we should identify this value and regard it as the threshold to determine the convergence. However, this value is uncontrollable because it can be affected by several factors, such as the number of subnetworks, random initialization, and training datasets. To mitigate this problem, we considered the difference between *kl_k_* and *kl_k−1_*. If this difference equals zero, it implies that there is no difference between *kl_k_* and *kl_k−1_*; moreover, it also implies that *kl_k_* converges.

However, it is challenging to use the difference between *kl_k_* and *kl_k−1_* as the criterion because empirically this difference becomes zero after a few iterations. The reason why this is problematic is that it leads to the shortage of training time for each subnetwork; this results in inaccurate imputation. Hence, we adopted the exponential moving average of the difference to ensure sufficient training time for the subnetworks. The exponential moving average at the *k*th iteration, *ema_k_*, is calculated according to Equation (7).

(7) $$ema_{k}=\alpha \left(kl_{k}-kl_{k-1}\right)+\left(1-\alpha \right)ema_{k-1}\;(k=2,3,\ldots ,N)$$

Here, α ∈ [0, 1] is a coefficient for how the latest value affects the exponential moving average. If *ema_k_* becomes zero, the training iteration will be completed. When *α* is large, the iterations will be completed early. Conversely, the required iteration times may be closer to *N_MAX_*; in this case, it is possible to obtain a better accuracy than the former case. However, *ema_k_* may become zero after a few iterations if *N* is small. This results from the large variations in the ensemble weights at early iterations. In this case, *α* should be larger or the minimum number of iterations should be set to prohibit an early conclusion. In the experiments, we set *α* to 0.025.

## 3. Results

### 3.1. Experimental Setup

We used the actual electric energy consumption data of a private university in Seoul, South Korea in our experiment. The data were collected every hour from 9 January 2015 to 28 February 2018 and were measured by dividing the campus into three building clusters depending on the usage and location of the buildings. The first cluster consisted of 32 academic buildings. The second cluster contained 16 residential buildings, and the third cluster consisted of five research buildings. The first cluster used 2656.26 kWh per hour on the average, and the minimum and maximum electric loads were 413.28 and 6835.20 kWh, respectively. In the case of the second cluster, the average, minimum, and maximum of energy load per hour were 1174.47, 467.64, and 2184.12 kWh, respectively. Lastly, the third cluster used 2656.26 kWh per hour on average, and the minimum. and maximum electric loads were 678.86 and 3502.20 kWh, respectively.

Moreover, we collected the weather and calendar data. The weather data, such as temperature, humidity, and wind speed, were provided by the Korea Meteorological Administration (KMA). Moreover, THI and WCI were calculated from the collected data. Using the calendar data, we determined the schedule of the university and the list of holidays on the web. As a result, 27,528 data points in each cluster were used in the experiments.

All experiments were implemented in Python using several libraries, such as Tensor-Flow [28], scikit-learn [29] and extreme gradient boosting (XGBoost) [30]. In the experiments, we intentionally deleted a portion of the dataset to simulate the existence of missing data points, and we used the remaining data as the training data, while the deleted data were used as test data to measure the accuracy. We denote the percentage of the amount of deleted data to the number of total data as *β*. For the accuracy comparison, we used the mean absolute percentage error (MAPE) and root mean square error (RMSE). MAPE and RMSE can be defined using Equations (8) and (9), respectively. Here, *A_t_* and *F_t_* are the actual and prediction values, respectively, and *n* is the number of data used in a test.

(8) $$MAPE=\frac{100}{n}\sum_{t=1}^{n}\left|\frac{A_{t}-F_{t}}{A_{t}}\right|$$

(9) $$RMSE=\sqrt{\frac{\sum_{t=1}^{n}{\left(A_{t}-F_{t}\right)}^{2}}{n}}$$

### 3.2. Comparison with Other Imputation Methods

To compare the proposed scheme with other imputation methods, we considered diverse imputation methods such as LI, HA, OWA, autoencoder-based imputation (AE), and generative adversarial imputation nets (GAIN) [31], and performed the comparison. In the case of the HA method, we utilized the available data from the electric energy consumption data corresponding to days of the same week, a previous time period, a subsequent time period, and the same time in previous years, which corresponds to the information presented in [7]. For the OWA method, we used the same setting as the HA method, and the hyperparameter of the OWA, *α*, was set as 0.2. The AE had three hidden layers each for its encoder (48–24–12 nodes) and for the decoder (12–24–48 nodes), and both generator and discriminator of GAIN were composed of three layers (48 nodes in each layer). Since both AE and GAIN required a multi-dimensional variable as their input, we cropped the energy consumption data to make them 48-dimensional inputs. Their batch sizes were set to 100, and the activation function was a rectified linear unit (ReLU) [32]. We used the adaptive moment estimation method (ADAM) as an optimizer [33] with a learning rate of 0.001, and trained them for 10,000 epochs. In the proposed scheme, each MLP consisted of four hidden layers (we followed the experimental results presented in Appendix A) and the number of nodes in each layer was 16, which was two-thirds of the number of the input variables [34,35]. The batch size, activation function, and optimizer were the same as the previous method. Moreover, L2 regularization was applied with *α* = 0.0001 to all MLPs, and dropout was not applied owing to degrading accuracy. *Epoch_enh_*, *Epoch_adj_*, and *N_MAX_* were set to 50, 50, and 200, respectively. Further, *N* was 20, and *α_th_* was 0.001.

In this experiment, we intentionally removed the *β* percentage of the dataset and imputed the deleted values with each method. While removing the data, we added certain missing intervals, such as a few hours or more. Using the imputed results, we calculated MAPE and RMSE; Table 2 lists these results. In the tables, for convenience, we refer to the proposed scheme as SENet.

It can be observed that most methods tend to show worse accuracy as the missing proportion (that is, *β*) increases. However, the decline in the accuracy of the proposed scheme is marginally small when compared to the other methods. This result is obtained as the proposed method is primarily based on the explanatory variables rather than the data points surrounding the missing data point. When the missing proportion increases, the time interval between the missing data point and the closest available data points are more significant, which results in the inaccuracy of LI. In the case of the HA method, the shortage of the historical data necessary for predicting the missing data points might cause inaccuracy. The OWA method appeared to be more robust than the other two methods because it is a combination of these two methods. Unlike these methods, the proposed method infers the missing data based on the explanatory variables. Thus, it can avoid the error increase even though several energy consumption data points are missing. Meanwhile, the results of AE and GAIN demonstrated that both models were not suitable for the energy consumption data we used.

In most cases, the proposed scheme achieved the lowest MAPE and RMSE. Excluding the proposed scheme, the OWA method exhibited better performance than the other two methods. In Clusters 1 and 3, the difference between each method in terms of both MAPE and RMSE was significantly demonstrated. Conversely, in Cluster 2, the difference between the OWA and the proposed scheme was marginal. The OWA showed the lowest MAPE, while the proposed scheme demonstrated the lowest RMSE. Thus, the OWA method demonstrates improved predictions for low actual values, and the proposed method demonstrates improved predictions for high actual values.

### 3.3. Comparison with Other Machine Learning Algorithms

For the verification of the ensemble model, SENet, we compared it with other machine learning algorithms that are used frequently in regression models. As the objective of this experiment is the performance of SENet, the input variables of each algorithm were the same. For comparison, we considered many popular machine learning algorithms, which include LR, RF, adaptive boosting (AD), gradient boosting machine (GBM), XGBoost, support vector regression (SVR), single MLP (MLP-S), convolutional neural network (CNN), recurrent neural network (RNN), and ensemble model adopting the average of subnetworks (MLP-AVG). Further, we considered our SENet with CNNs and RNNs. To measure each accuracy, we conducted a five-fold cross-validation and calculated the mean of the errors.

The hyperparameters of the algorithms were set by a grid search except for the deep learning-based algorithms. As the training of deep learning is significantly time consuming, we did not consider the grid search. Instead, we set the hyperparameters of the MLPs identically. The details of MLP and SENet are already described in the previous subsection. Thus, we have omitted an explanation of their settings. In the case of CNN and RNN, we merged and utilized input variables of six available consequent time steps because they demanded a sequence of input variables as their input. The CNN consisted of four convolutional layers (32 filters in each convolutional layer) and two fully-connected layers (32–16 nodes), and the RNN had two long short term memory (LSTM) layers (25 dimensions) and two fully-connected layers (25–12 nodes). For the RNN, we adjusted the learning rate to 0.01 for fast training. These structures were also used in the SENet with CNN and RNN. Other hyperparameters of the RNN and CNN were the same as those of the MLPs or SENet. 

Table 3 lists the experimental results. SENet showed superior prediction performance in most cases. The MAPE of RF, commonly used in electric load forecasting [36,37], was similar to the MAPE of SENet. However, in terms of RMSE, SENet achieved significantly lower errors than RF. MLP-AVG showed a comparable prediction performance. However, it required much longer training times and larger memory than the SENet because it used all subnetworks, while it showed marginally larger errors. Hence, the SENet is superior to MLP-AVG. Meanwhile, the CNN and RNN did not show better performances than MLP-S. The SENet with CNN or RNN performed better than CNN or RNN, but did not catch up with our SENet with MLPs. Thus, we used MLP instead of CNN or RNN to construct our SENet. 

### 3.4. Impact of the Number of Subnetworks

In this experiment, we examined the proposed scheme with the same conditions described in the previous subsection, except for the number of subnetworks. We set the numbers of subnetworks to 10, 20, 40, and 60 sequentially, and the results are listed in Table 4.

As the number of subnetworks increased, the imputation errors became lower. However, the reduced amount of the errors was marginal, considering the dramatic increase in training time owing to the use of several subnetworks. Nevertheless, when a significantly accurate missing-value imputation is essential regardless of training times and memory consumption, setting a large number of subnetworks is one of the reasonable choices.

### 3.5. Impact of α_th_

This experiment was conducted for confirming the impact of *α_th_*. The experimental setting was the same as that described in Section 3.3; however, *α_th_* ranged from 0.001 to 0.1. The dataset used was the energy consumption data of Cluster 3, and we measured MAPE and RMSE. Furthermore, we counted the remaining subnetworks and calculated the remaining ratio. These results are listed in Table 5.

Regardless of *α_th_*, MAPE and RMSE were approximately consistent without any noticeable tendency. However, in terms of the remaining ratio, a significant difference can be observed. When *α_th_* was set high, the subnetworks were removed frequently, which resulted in the compressed ensemble model. However, if *α_th_* was set higher than one over *N* (in this experiment, it was 0.05), we observed that the number of the remaining subnetworks was close to one and the errors were increased. This implies that the trained model was a single MLP rather than an ensemble model. Hence, we recommend that *α_th_* should be lower than one over *N* to demonstrate the ensemble effect.

### 3.6. Immediate Check

**Figure 6:**
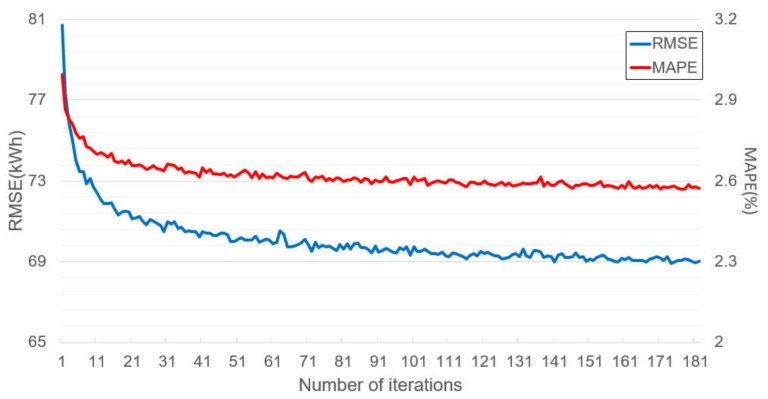
Variations of test error in SENet during training.

**Figure 7:**
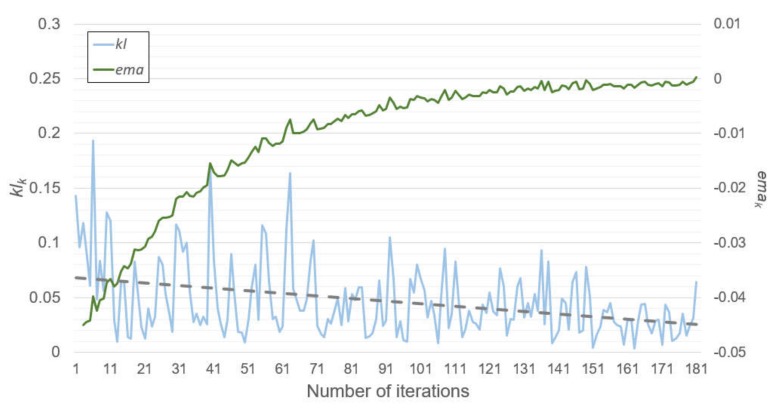
Variations of *kl_k_* and *ema_k_* in SENet during training.

To show the variations observed during the model training, we recorded the test errors, *kl_k_* and *ema_k_* at each iteration. Figure 6 and Figure 7 show these results, respectively. These results were obtained from when the SENet was trained by the dataset from Cluster 1, and the experimental setting was the same as the previous experiments. Moreover, the model training was completed after the 181st iteration owing to the convergence test.

Figure 6 and Figure 7 represent the test error changes and *kl_k_* and *ema_k_* changes at each iteration, respectively. At early iterations, the test errors rapidly decreased. At the same time, *kl_k_* varied from approximately 0 to 0.2. However, the test errors and the *ema_k_* started to converge in the middle of the iterations, while the range of the *kl_k_* variations was reduced, and the *kl_k_* tended to become small according to its trend line. After the 181st iteration, the *ema_k_* became zero, and the model training was concluded. It can be observed from Figure 6 that there were only a few benefits in terms of accuracy errors or the number of subnetworks, even if the model training continued.

## 4. Conclusions

In this study, we proposed a missing-value imputation method using an ensemble scheme based on MLPs and several explanatory variables. To this end, we collected additional data and constructed the dataset for training the ensemble model. Then, we used random sampling with replacement and the softmax function to improve the ensemble effects. For proving the effectiveness of the proposed scheme, we compared our scheme with other missing-value imputation methods and machine learning algorithms. We confirmed that the proposed scheme delivered superior performance.

In future works, we shall attempt to combine two training stages into one for realizing a simple training process and shorter training time that can aid the implementation of the missing-value imputation in real-time environments. Further, we plan to develop a method to reduce the number of input variables used while maintaining a superior imputation performance.

## Figures and Tables

**Table 1:** List of the input variables and the output variable.

Class	Variable Name	Type [Range]	Variable Name	Type [Range]
Input Variable	*Month_x*	Continuous [−1, 1]	*Working_hour*	Integer [0, 1]
	*Month_y*	Continuous [−1, 1]	*Monday*	Integer [0, 1]
	*Day_x*	Continuous [−1, 1]	*Tuesday*	Integer [0, 1]
	*Day_y*	Continuous [−1, 1]	*Wednesday*	Integer [0, 1]
	*Hour_x*	Continuous [−1, 1]	*Thursday*	Integer [0, 1]
	*Hour_y*	Continuous [−1, 1]	*Friday*	Integer [0, 1]
	*Minute_x*	Continuous [−1, 1]	*Saturday*	Integer [0, 1]
	*Minute_y*	Continuous [−1, 1]	*Sunday*	Integer [0, 1]
	*Spring*	Integer [0, 1]	*Temperature*	Continuous
	*Summer*	Integer [0, 1]	*Humidity*	Continuous
	*Fall*	Integer [0, 1]	*Wind*	Continuous
	*Winter*	Integer [0, 1]	*THI*	Continuous
	*Holiday*	Integer [0, 1]	*WCI*	Continuous
Output Variable	*Energy* *Consumption*	Continuous		

**Table 2:** Mean absolute percentage error (MAPE) and root mean square error (RMSE) comparison according to *β*. The values in parenthesis indicate RMSE, and the values in bold font indicate the lowest values for respective clusters.

*β*	Model	Cluster 1	Cluster 2	Cluster 3
10%	LI	8.780 (438.330)	6.235 (109.287)	4.496 (172.061)
	HA	9.713 (393.236)	7.475 (115.267)	3.759 (116.384)
	OWA	6.179 (244.755)	**5.567** (91.362)	2.666 (84.613)
	AE	11.929 (618.432)	8.555 (149.014)	5.621 (221.987)
	GAIN	12.899 (485.478)	8.231 (135.504)	5.552 (196.853)
	SENet	**4.704 (163.832)**	5.847 **(90.354)**	**1.817** (**52.455)**
15%	LI	10.984 (433.465)	6.376 (112.723)	4.589 (175.119)
	HA	10.037 (400.172)	7.465 (113.640)	4.144 (135.776)
	OWA	7.246 (273.693)	**5.530** (86.608)	2.907 (97.644)
	AE	11.758 (549.025)	8.643 (144.683)	5.396 (208.899)
	GAIN	14.519 (481.857)	8.108 (124.898)	5.448 (181.327)
	SENet	**5.162 (172.117)**	5.784 **(83.122)**	**2.018 (59.325)**
20%	LI	12.280 (479.293)	6.743 (112.875)	4.652 (159.807)
	HA	10.549 (412.181)	7.545 (112.965)	4.244 (131.663)
	OWA	8.221 (280.827)	5.794 (89.607)	3.033 (98.397)
	AE	11.104 (515.135)	8.363 (149.068)	5.010 (176.925)
	GAIN	14.173 (455.746)	8.024 (121.281)	5.792 (191.480)
	SENet	**5.212 (181.821)**	**5.792 (86.078)**	**2.056 (62.146)**
25%	LI	15.062 (527.053)	7.163 (121.697)	5.623 (210.698)
	HA	10.895 (428.504)	7.395 (113.157)	4.366 (146.488)
	OWA	8.601 (326.815)	5.773 (90.735)	3.311 (117.112)
	AE	12.722 (564.855)	8.589 (153.946)	5.085 (184.643)
	GAIN	14.575 (486.518)	8.032 (137.447)	5.601 (188.025)
	SENet	**5.370 (188.827)**	**5.861 (84.289)**	**2.092 (60.524)**
30%	LI	16.242 (575.417)	6.982 (120.153)	5.820 (218.499)
	HA	11.117 (423.496)	7.547 (115.421)	4.544 (145.974)
	OWA	9.876 (317.934)	**5.811** (92.514)	3.743 (126.125)
	AE	13.131 (590.977)	8.795 (158.521)	4.822 (182.089)
	GAIN	14.233 (453.511)	8.102 (130.569)	5.894 (191.993)
	SENet	**5.544 (188.013)**	5.836 **(85.928)**	**2.080 (62.576)**

**Table 3:** MAPE and RMSE comparison for various models. The values in parenthesis indicate RMSEs, and the values in bold font indicate the lowest values for respective clusters.

Model	Cluster 1	Cluster 2	Cluster 3
LR	21.681 (626.956)	12.925 (184.881)	7.252 (192.009)
AD	25.016 (561.639)	12.509 (163.882)	6.833 (169.560)
SVR	11.786 (354.800)	9.108 (132.927)	3.883 (111.193)
GBM	7.797 (249.126)	7.716 (112.051)	3.045 (83.570)
XGBoost	9.406 (318.314)	9.070 (131.006)	3.580 (100.494)
RF	5.456 (205.678)	6.601 (97.068)	**2.616** (75.053)
MLP-S	6.233 (206.313)	7.583 (110.229)	2.978 (79.445)
CNN	10.469 (379.371)	13.728 (206.747)	4.910 (124.361)
RNN	8.697 (308.594)	10.452 (155.490)	3.701 (106.469)
MLP-AVG	5.407 (182.942)	6.535 (94.092)	2.692 (73.202)
SENet with CNN	10.047 (319.160)	11.745 (175.581)	4.693 (125.920)
SENet with RNN	6.447 (222.212)	8.548 (124.782)	3.001 (84.523)
SENet with MLP (proposed)	**5.391 (181.988)**	**6.440 (93.384)**	2.658 **(70.858)**

**Table 4:** MAPE and RMSE comparison according to the number of subnetworks. The values in parenthesis indicate RMSEs and the values in the bold font indicate the lowest values for respective clusters.

*N*	Cluster 1	Cluster 2	Cluster 3
10	5.495 (186.537)	7.072 (102.090)	2.757 (73.432)
20	5.391 (181.988)	6.440 (93.384)	2.658 (70.858)
40	5.401 (180.132)	6.468 (93.601)	2.604 (69.258)
60	**5.252 (176.517)**	**6.327 (92.732)**	**2.583 (68.561)**

**Table 5:** MAPE and RMSE comparison according to the changes in *α_th_*. The values in bold font indicate the lowest MAPEs and RMSEs for respective clusters.

α_th_	MAPE (%)	RMSE (kWh)	Remaining Ratio (%)
0.1	2.989	80.001	5%
0.05	2.676	71.667	30%
0.01	**2.656**	**70.721**	54%
0.005	2.666	70.802	58%
0.001	2.687	71.534	69%

## Appendix A

We conducted experiments under the same conditions of the experiment in Section 3.2 (β = 20%) to decide the number of hidden layers in one MLP. We varied the number of hidden layers and measured both MAPE and RMSE of the test set. The following table shows the results. In the table, we can see that four hidden layers gave the best overall performance.

**Table A1:** Experimental results according to the number of hidden layers. The values in bold font indicate the lowest MAPE and RMSE for respective clusters.

Dataset	No. of Hidden Layers	RMSE	MAPE
Cluster 1	2	192.266	5.709
	3	**181.392**	5.366
	4	181.821	**5.212**
	5	182.314	5.217
Cluster 2	2	**85.444**	5.891
	3	85.612	5.883
	4	86.078	**5.792**
	5	88.500	6.006
Cluster 3	2	64.748	2.142
	3	63.911	2.105
	4	**62.146**	**2.056**
	5	63.342	2.079
